# Surveillance of Environmental and Procedural Measures of Infection Control in the Operating Theatre Setting

**DOI:** 10.3390/ijerph15010046

**Published:** 2017-12-28

**Authors:** Laura Dallolio, Alessandra Raggi, Tiziana Sanna, Magda Mazzetti, Alessandra Orsi, Angela Zanni, Patrizia Farruggia, Erica Leoni

**Affiliations:** 1 Department of Biomedical and Neuromotor Sciences, Unit of Hygiene, Public Health and Medical Statistics, University of Bologna, via S. Giacomo 12, 40126 Bologna, Italy; alessandra.raggi2@studio.unibo.it (A.R.); t.sanna@ausl.bologna.it (T.S.); erica.leoni@unibo.it (E.L.); 2 Unit of Hygiene, Control of Healthcare Associated Infections, Local Health Authority of Bologna, Bellaria Hospital, via Altura 3, 40139 Bologna, Italy; magda.mazzetti@ausl.bologna.it; 3 Unit of Hygiene and Quality of Residential Services, Local Health Authority of Bologna, Bellaria Hospital, via Altura 3, 40139 Bologna, Italy; a.orsi@ausl.bologna.it (A.O.); angela.zanni@ausl.bologna.it (A.Z.); patrizia.farruggia@ausl.bologna.it (P.F.)

**Keywords:** surgical site infections, operating theatres, surgery cleaning procedures, microbiological contamination, evidence-based surgical good practices

## Abstract

The microbiological contamination of operating theatres and the lack of adherence to best practices by surgical staff represent some of the factors affecting Surgical Site Infections (SSIs). The aim of the present study was to assess the microbiological quality of operating settings and the staff compliance to the SSI evidence-based control measures. Ten operating rooms were examined for microbiological contamination of air and surfaces, after cleaning procedures, in “at rest” conditions. Furthermore, 10 surgical operations were monitored to assess staff compliance to the recommended practices. None of the air samples exceeded microbiological reference standards and only six of the 200 surface samples (3.0%) were slightly above recommended levels. Potentially pathogenic bacteria and moulds were never detected. Staff compliance to best practices varied depending on the type of behaviour investigated and the role of the operator. The major not compliant behaviours were: pre-operative skin antisepsis, crowding of the operating room and hand hygiene of the anaesthetist. The good environmental microbiological quality observed is indicative of the efficacy of the cleaning-sanitization procedures adopted. The major critical point was staff compliance to recommended practices. Awareness campaigns are therefore necessary, aimed at improving the organisation of work so as to facilitate compliance to operative protocols.

## 1. Introduction

Surgical Site Infections (SSIs) are associated with virtually any surgical procedure and represent one of the main complications in surgical patients [1]. They are the second most frequent type of Healthcare Associated Infection (HAI) in Europe and the United States [2].

SSIs are complex and multifactorial events and many factors have been identified as contributing to the risk of their occurrence. These factors can be patient-related and process/procedural-related as well as depending on the level of environmental microbial contamination in the operating room [2]. The extent to which each of these factors can contribute to infection onset is unknown, but it is certain that the majority of SSIs are caused by the patient’s endogenous flora and are acquired in the operating room, during surgical procedures, when microorganisms could reach the open wound [3]. SSIs result in a significant increase in morbidity and mortality in surgical patients and, among all HAI, they represent those with the greatest economic and financial impact, due to a longer duration of postoperative hospitalization, and an increased risk of readmission, additional surgical procedures and admission to intensive care units [4,5,6]. Considering their negative impact, reducing the rate of SSIs is imperative. It has been estimated that up to 50% of SSIs could be avoided through the implementation of adequate preventive evidence-based strategies [5]. Environmental cleaning and disinfection procedures, together with the implementation of proper behavioural practices, are essential in reducing microbial contamination and the incidence of nosocomial infections [7].

This study aimed to assess some of the process/procedural-related factors conditioning the SSI risk, particularly the effectiveness and proper application of cleaning and sanitization protocols in operating settings, by determining the level of microbiological contamination of air and critical surfaces. Surgical staff compliance to the evidence-based prevention measures was also assessed.

## 2. Materials and Methods

### 2.1. Setting

The operating blocks of two health facilities in Bologna (Emilia Romagna, Northern Italy) were examined. All the operating theatres had the same structural characteristics and organization of physical spaces. They were ventilated with a turbulent airflow system providing a positive pressure and 15 air changes/hour. The same written operative protocol (concerning environmental sanitization, reconditioning of surgical instruments, preparation of patients, antibiotic prophylaxis, behavioural norms) was applied in each operating room.

Operating room cleaning practices were carried out by competent and trained personnel between each operation and at the end of the daily operating session. Between one operation and the next, the cleaning protocol provides in sequence: (i) removal of waste from the designated containers; (ii) removal and disinfection (10% sodium hypochlorite) of biological materials (blood, faeces, vomit, etc.) from visibly contaminated surfaces and (iii) cleaning and sanitizing of all surfaces. In accordance with the generally accepted principles, surface cleaning starts from the cleanest area to the dirtiest and from the highest level to the lowest. All the surfaces which come into contact with patients’ blood or body fluids (e.g., operating table) and high hand-touch surfaces (e.g., surgical lighting, touch screens, monitors, tables, trolleys, button panels, etc.) are treated with a detergent (non-ionic tensioactive) and disinfectant (active chlorine 1080 ppm) solution for an action time of 5 min. Lastly, the floor is washed with a detergent and disinfectant solution (active chlorine: 540 ppm).

At the end of daily activity, cleaning procedures are repeated in the same manner. In addition, all mobile furnishing and equipment is taken outside the operating room and all the surfaces, including those not coming into contact with the patient and the hands of the operators (walls, aeration devices, shelves, fixed equipment, floors) are cleaned. Then, after being cleaned and disinfected, the mobile furnishings are taken back into the operating room. For the end-of-day cleaning a concentration of 540 ppm active chlorine is used.

### 2.2. Microbiological Sampling

A total of 10 operating rooms were examined (four in Hospital A and six in Hospital B). Sampling was carried out “at rest”, when no patients and surgical staff were in the operating theatre; two moments of the same daily operating session were chosen: (i) Pre: at the beginning of each day, before the first scheduled operation, when the operating rooms had been sanitized during the previous evening; (ii) Post: before the second daily scheduled operation, immediately after a clean environment had been re-established.

For each operating room, air and surfaces were sampled for microbiological analysis. Ten surfaces were identified as “critical”, as more frequently touched: medical anaesthesia trolley, nurse’s computer touch screen, operating table, vitals monitor, anaesthetist’s computer touch screen, surgical lighting, instrument table, operating table remote control, internal door opening button, and operating theatre intercom [8,9,10].

Active air sampling was carried out according to UNI EN ISO 13098, 2002, using the Surface Air System (SAS) sampler (VWR PBI International, Milan, Italy). The air aspirated by the SAS was impacted onto the surface of an agarized growth medium specific for the microorganisms under investigation. Sampling took place in the centre of the room, at about 0.5 m from the operating table and 1.5 m from the floor.

Sampling of surfaces was carried out in two different ways, depending on the surface examined. On regular and flat surfaces, the contact plate method was chosen, using RODAC (Replicate Organism Direct Agar Contact) imprint technique (UNI EN ISO 14698-1, 2004). RODAC plates (diameter: 55 mm; contact surface: 24 cm^2^) were pressed onto the surfaces for 10 s, applying a constant pressure. To collect environmental samples from irregular surfaces, sterile calcium alginate swabs (SRK 10 mL 21903C, Italian Biolife SDA, Milan, Italy) were rubbed and rolled firmly across the previously measured sampling area, using parallel and close scrapes. Each swab was then placed into tubes with 10 mL of transport liquid (Ringer’s solution). A total of 200 samples were obtained (RODAC plates: 140; swabs: 60). At the end of sampling, plates and swabs were placed in refrigerated containers and were transported to the laboratory for microbiological analysis.

### 2.3. Microbiological Analysis

Total Viable Count (TVC) was determined on Plate Count Agar (PCA, Italian Biolife SDA). PCA plates obtained by SAS, RODAC and by inoculating the swab transport liquid were incubated at 36 °C for 48 h. All colony forming units (cfu) on PCA were counted and subcultured in order to identify *Staphylococcus aureus*, *Enterobacteriaceae*, Enterococci and *Pseudomonas* spp. by morphological and biochemical features (API miniaturized biochemical tests, bioMérieux, Marcy l’Etoile, France). Total Mould Count (TMC) was determined on Sabouraud Dextrose Agar (Italian Biolife, SDA). Following an incubation time of 5–7 days at 25 °C, SDA grown moulds were macroscopically and microscopically examined to exclude the presence of *Aspergillus* spp. For the air counts, the number of colony forming units was adjusted using the conversion table provided by the SAS manufacturer and was expressed in cfu/m^3^. Results for surfaces were expressed as cfu/plate (RODAC) or cfu/cm^2^ in the case of swab sampling.

### 2.4. Standards for Microbiological Indicators

The Italian Istituto Superiore Prevenzione e Sicurezza del Lavoro (ISPESL) Guidelines were taken as reference [9]: in the vicinity of the operating table, in “at rest” conditions, the guidelines recommend an air contamination limit of TVC ≤ 35 cfu/m^3^, already proposed by the HTM 2025 [11]. For surface contamination the same Italian guidelines suggest referring to the limits proposed in the 1999 French Guidelines [12] and confirmed by the recent 2016 French Guidelines [13]. On these bases, the TVC in the hand-touch sites of the operating rooms should not exceed 5 cfu/plate (expected value), while values >5 and ≤15 cfu/plate are considered acceptable, and TVC > 15 cfu/plate in more than one surface sampled in the same operating room indicates hygiene failure. For the samples collected by swabbing a limit of TVC ≤ 1 cfu/cm^2^ was considered acceptable. *S. aureus*, *Enterobacteriaceae*, *Pseudomonas* spp., *Aspergillus* spp. must always be absent.

### 2.5. Compliance to Best Practice

A total of 10 surgical operations were monitored, on the same days and in the same operating rooms chosen for microbiological analysis. Compliance to best practices was assessed by referring to the main national [14] and international [3,4,15,16] guidelines and using a structured and purpose-made observation grid. Table 1 shows the procedures and behaviours that were monitored.

### 2.6. Statistical Analysis

Statistical analyses were performed using the SPSS software, version 20 (IBM SPSS Statistics, IBM Corporation, Armonk City, NY, USA). The microbiological data (cfu) were converted into Log_10_ (x + 1) to normalize the non-normal distributions. The results are presented as geometric means with confidence intervals. ANOVA was used to compare the difference between means. A *p* value < 0.05 was considered as statistically significant. Compliance to behavioural norms was assessed by calculating the percentage frequencies of the behaviours observed.

## 3. Results

### 3.1. Microbiological Air Quality

In both samplings of the day (Pre- and Post-) the microbial contamination of the air was always within the limit of 35 cfu/m^3^ (range: 2–27 cfu/m^3^) established by the ISPESL Italian Guidelines for conventional operating theatres at rest [9]. *S. aureus*, *Enterobacteriaceae*, Enterococci, *Pseudomonas* spp. were never detected. The identified species (around 50% of isolates) belonged mainly to the *Staphylococcus* genus: *S. epidermidis* (21.8% of isolates), *S. hominis* (6.3%), *S. xylosus* (3.6%), *S. warneri* (2.8%). The hyphomycete isolates (four isolates in all) belonged to the *Cladosporium* genus.

### 3.2. Microbiological Quality of Surfaces

Table 2 shows the compliance of samples to the reference values, the geometric mean and confidence intervals (CI 95%) of TVC in the surfaces monitored at rest. With reference to the standards of the Italian ISPESL Guidelines (acceptable value ≤ 15 cfu/plate), the TVC by RODAC method exceeded the acceptable limit only in 3 (2.1%) of the sampled surfaces. The non-conforming surfaces were: one anaesthetist’s computer touch screen (TVC: 16 cfu/plate); two surgical lighting fixtures (16 and 41 cfu/plate respectively). Another three surfaces sampled using swabs (5.0%) exceeded the acceptable value (≤1 cfu/cm^2^): two internal door opening buttons (1.7 and 3.1 cfu/cm^2^ respectively) and one intercom (3.0 cfu/cm^2^). Overall, in 40.5% of samples the TVC was 0 and in a further 56.5% conformed to standards. The surfaces exceeding the acceptable value were in six different operating rooms. Bacteria such as *S. aureus*, *Enterobacteriaceae*, Enterococci, *Pseudomonas* spp. were never detected. No moulds were even isolated. The bacteria species identified (around 70% of isolates) mainly belonged to the *Staphylococcus* genus: *S. epidermidis* (28.2% of isolates), *S. hominis* (12.0%), *S. xylosus* (10.3%), *S. haemolyticus* (8.5%), *S. warneri* (7.7%), *S. cohnii* (4.3%).

As far as the Pre- and Post- samplings are concerned (RODAC sampling), the number of samples exceeding the TVC expected values is higher in those taken before the first operation (Pre), and a similar situation was found for the mean counts, although the differences were not statistically significant (geometric means: 2.0 vs. 1.6; ANOVA, *p* = 0.07). Comparing the two hospitals, the mean value of TVC and the number of samples exceeding the expected value (RODAC sampling) were higher in hospital A than in hospital B, with statistically significant differences (geometric means: 2.2 vs. 1.5; ANOVA, *p* = 0.01). These differences are mainly due to the results in the Pre sampling (Table 2).

Table 3 shows the geometric means of TVCs in the various sampled surfaces. The values can be compared only in the context of the same sampling technique. The values obtained using the swab method should be considered with due caution, as this technique is applicable as a quantitative method for surfaces with TVC > 100 cfu/cm^2^ [17]. In the low contaminated surfaces of this study the technique was used prevalently to exclude the presence of pathogenic microorganisms. Among the surfaces sampled with the RODAC method, the highest TVC mean values were recorded in: the scialytic lamp (2.6 cfu/plate), the instrument table (2.4 cfu/plate) and the medical anaesthesia trolley (2.1 cfu/plate). The internal door opening button was the most contaminated surface among those sampled with a swab, both in the number of samples exceeding the expected value, and as a mean (10.6 cfu/24 cm^2^).

### 3.3. Compliance to Best Practice

The observations on conditions and behaviours were made during 10 operations of general surgery (two laparoscopic cholecystectomies, one inguinal hernia, one mastectomy), otolaryngologic surgery (two thyroidectomies), urology (one transvescical adenomectomy), neurosurgery (one craniotomy, one spinal canal stenosis laminectomy), orthopaedics (one knee prosthesis). 

With regards to the operating conditions, the main lack of compliance was the crowding of the room, mainly due to the entrance of personnel not related to the operation (Table 4). The number of people present in the operating room at the same time was from 6 to 12 (mean 9) e in all the cases there was a traffic flow due to people not related to the operation. Another non-compliant behaviour was the incorrect performance of the pre-operative skin antisepsis which, in seven operations out of 10, was carried out without waiting for the spontaneous drying of the antiseptic, which was used in excessive quantities and dried with a sterile fabric. In some cases, a non-centrifugal technique was also used.

Regarding the staff, 122 professional healthcare workers were observed, 84 of whom were directly related to the operation and 38 not related (Table 5). Among the first group, 74 belonged to the surgical teams (surgeons, scrub nurses, anaesthetists, operating room nurses), and 10 were trainee doctors and nurses, anatomo-pathologists or specialists. Those unrelated to the operation were anaesthetists and nurses from adjacent operating rooms who came to ask for information. All the observed staff wore clothing specific to the operating room, while around 65% correctly used caps and masks. The surgeons and scrub nurses showed the greatest compliance to dress codes (non-compliance: 10.5%), while the staff not related to the operation showed less attention than those who were directly involved (52.6% vs. 27.4%). Hand washing and sterile clothing (compulsory for surgeons and scrub nurses) was not adhered to by one operator out of 38. In addition, 22 (57.9%) did not wash their hands after removing the sterile gloves; non-compliance was more frequent among surgeons than scrub nurses (79.2% vs. 21.4%). Another not compliant behaviour was the failure of anaesthetists to wash their hands before any contact with the patient; this procedure was never followed in 46.2% and only sometimes in the remaining 53.8%.

## 4. Discussion

The microbiological quality of the air and surfaces of the operating theatres examined in at rest conditions was good in both hospitals. None of the air samples exceeded the TVC reference standards and only six of the 200 surface samples (3.0%) showed slightly higher values. Potentially pathogenic bacteria and moulds were never detected in the surfaces or in the air. The findings of the study are indicative of the efficacy of the cleaning-sanitization procedures adopted, as well as of the appropriate characteristics of the ventilation system which plays a fundamental role in maintaining a low microbial contamination level in operating rooms [18,19].

The surfaces were less contaminated in the samplings made in the operating rooms after being prepared for the second operation. This indicates that the cleaning-sanitization procedures foreseen between one operation and the next, if performed correctly, are effective in reducing the contamination to very low levels, lower than those of the room at the start of the day. Subsequent to the results of our study, supplementary cleaning was introduced in the morning, before the start of the session, to further reduce the levels of contamination, as recommended in some guidelines [2,8]. With regards to the various types of surface investigated, the scialytic lamp (RODAC method) and the internal door opening button of the operating theatre (swab sampling) had higher mean TVC values and also exceeded the reference limits in two cases (10% of samples both for scialytic lamp and opening botton). On account of its position, the scialytic lamp is a particularly critical surface, being situated between the air flow (above) and the operating table (below); therefore, any impurities present on its surface can be projected onto the operating area by the flux of air.

As far as the staff behaviour is concerned, the degree of compliance to the post-operative infection prevention measures differed depending on the particular procedural measure observed and the type of professional role. A good level of compliance was observed for the antibiotic-prophylaxis and the preparation of the surgical team (surgical hand-washing and sterile dress for surgeons and scrub nurses), which appear to be well consolidated practices. In general, the staff whose protocol foresees surgical hand-washing and sterile dress (surgeons and scrub nurses), were more compliant to the recommended practices, better among the scrub nurses than the surgeons. The major inappropriate behaviours concerned the pre-operative skin antisepsis, the crowding of the operating rooms and the hand hygiene of the anaesthetists before contact with the patient.

With respect to the pre-operative skin antisepsis, the most frequent non-compliance was the use of excessive quantities of the antiseptic, applied with a non-centrifugal technique and dried with a sterile fabric. There is a lack of conclusive evidence on the optimal preoperative technique for skin preparation, regarding centrifugal method or back and forth motion [20]. In this study the centrifugal technique was considered correct on the basis of skin antiseptic manufacturers’ instructions.

As regards the crowding of the operating room, this was due to the entrance of personnel unrelated to the operation, who also appear less attentive to best practices and are therefore more likely to contribute to the environmental contamination. Similar results about traffic flow during surgical procedures were described by Loison et al. [21]. Various studies showed that the number people present and the frequency of door opening can lead to an increase in the level of bacteria and airborne particles [22,23,24]. Crowding of the operating room increases the risk of accidental contamination of sterile areas and instruments, and may lead to the distraction of the surgeon, increasing the possibility of errors. Moreover, the frequent opening of doors compromises the performance of the ventilation system in controlling environmental contamination [25,26,27,28]. A limitation to our study is that it does not investigate the level of microbial contamination in “operational” conditions. As a result, it was not possible to assess the impact of crowding and the frequent opening of the doors on the quality of the air.

An important lack of compliance was observed in the anaesthetists’ hand-washing. Despite being a well-known and fundamental measure for reducing the risk of infection in all health settings, it was never performed correctly in any of the operations observed. The availability of an antiseptic product to disinfect the hands without water might encourage greater compliance to this aspect.

Similar critical behaviours emerged from regional data on SSI control measures [29]. According to this dossier, surgeons believe that one of the main reasons preventing full compliance to the recommended measures is a lack of culture, training and development; nurses, on the other hand, place more blame on a lack of resources and on organisational and management issues. Another obstacle is the failure of operators to perceive any difference between their own practices and the recommendations. Moreover, they mistakenly tend to place greater importance on environmental control, underestimating the importance of good behavioural norms [29].

The operations monitored in our study are all included in the regional surveillance plan of SSIs, whose purpose is to monitor a representative sample of the surgical operations performed throughout the whole region and to assess the incidence and the distribution of infections in the surgical sites. The data from the regional surveillance system showed that no infections were detected for the 10 surgical operations investigated in this study. However, the number of operations monitored is too limited to draw any conclusions.

## 5. Conclusions

The results of this study underline the importance of surveillance, regarding both environmental measures and, more especially, behavioural measures, where some critical areas emerged. The instances of non-compliance to the environmental standards highlight the need for great attention in cleaning procedures and improvement of the protocols previously adopted. The monitoring of adhesion to cleaning-sanitization procedures and behavioural norms can provide findings that should be communicated and discussed with the personnel involved, thus raising their awareness. Also the improvement of work conditions could facilitate compliance to prevention measures and operative protocols.

## Figures and Tables

**Table 1:** Infection prevention measures undertaken to surveillance.

Pre-Operative Measures	Intraoperative Measures	Post-Operative Measures	Other Observations
hair removal: only when necessary, using a single use electric or battery-operated clipper, as close as possible to the moment of operation, but not in the operating room	crowding of operating room: number of people unrelated to the operation entering to the operating room	asepsis/aseptic technique in the final medication: final dressing must be performed without touching the surgical wound	use of appropriate operating room attire
appropriate antibiotic: appropriate indications and molecule; timing: within 1 h from incision	doors closed during operation	hand hygiene in surgeons and scrub nurses after removing gloves	presence of personal objects in the operating room
pre-operative skin antisepsis: preparation of skin with chlorhexidine or iodophors in alcohol-based solution, centrifugal technique, spontaneous drying of product	correct use of cap/haircover (completely containing hair and beard)		hand hygiene in anaesthetist before contact with patient
correct surgical hand-washing (for 3–5 min, with use of antiseptic, removal of jewellery) and sterile attire in surgeons and scrub nurses	correct use of mask (completely covering nose and mouth)		

**Table 2:** Total Viable Count (TVC) in the surfaces of the operating rooms monitored “at rest”, in two moments of the same daily operating session (Pre and Post). Distribution of samples in the different contamination classes, geometric means and confidence intervals (CI 95%).

	Pre				Post			
Operating Rooms	RODAC Sampling ^a^		Swab Sampling ^b^		RODAC Sampling ^a^		Swab Sampling ^b^	
	N: 7		N: 3		N: 7		N: 3	
	N. of Samples with cfu/plate		N. of Samples with cfu/cm^2^		N. of Samples with cfu/plate		N. of Samples with cfu/cm^2^	
	≤5	5 < x ≤ 15	>15	>1	≤5	5 < x ≤ 15	>15	>1
1A	5	1	1	0	7	0	0	1
2A	5	2	0	0	7	0	0	0
3A	6	1	0	0	6	0	0	1
4A	7	0	0	1	7	0	1	0
Geometric mean (CI 95%)	3.1 (2.2–4.3)		1.5 (1.1–2.0)		1.5 (1.2–2.1)		1.4 (1.1–1.9)	
1B	6	0	1	0	6	1	0	0
2B	7	0	0	0	6	1	0	0
3B	7	0	0	0	7	0	0	0
4B	7	0	0	0	7	0	0	0
5B	7	0	0	0	7	0	0	0
6B	7	0	0	0	7	0	0	0
Geometric mean (CI 95%)	1.5 (1.2–1.8)		1.3 (1.2–1.5)		1.6 (1.3–2.0)		1.3 (1.1–1.5).	

^a^ Expected value: TVC ≤ 5 cfu/plate; acceptable values: TVC 5 < x ≤ 15 cfu/plate; alert value: TVC > 15 cfu/plate; ^b^ acceptable value: TVC ≤ 1 cfu/cm^2^.

**Table 3:** Geometric mean, confidence intervals (CI 95%) of Total Viable Counts (TVC) in the surfaces monitored “at rest”. Comparison of the different sampled surfaces.

Sampling Method	Sampled Surfaces (Sampled Area)	Compliance with Expected Values ^a^ N. (%)	TVC (cfu/24 cm^2^) ^b^	
			Geometric Mean	CI 95%
RODAC (sampled area: 24 cm^2^)	medical anaesthesia trolley	19 (95%)	2.1	1.4–3.0
	nurse’s computer touch screen	20 (100%)	1.1	1.0–1.4
	operating table	18 (90%)	1.7	1.2–2.5
	vitals monitor	20 (100%)	1.4	1.1–1.7
	anaesthetist’s computer touch screen	18 (90%)	1.6	1.1–2.4
	surgical lighting	18 (90%)	2.6	1.6–4.1
	instrument table	18 (90%)	2.4	1.7–3.4
Swab (the whole surface was sampled)	operating table remote control (75 cm^2^)	20 (100%)	3.8	2.7–5.2
	internal door opening button (49 cm^2^)	18 (90%)	10.6	6.3–17.7
	operating theatre intercom (36 cm^2^)	19 (95%)	7.0	4.0–12.2

^a^ expected value: TVC ≤ 5 cfu/plate (RODAC); TVC ≤ 1 cfu/cm^2^ (Swab); ^b^ TVC referred to 24 cm^2^ to compare the results obtained with the two sampling methods.

**Table 4:** Compliance to best practice. Observations regarding the operation.

Procedure	Not compliant Operations N. (%)	Non-Compliance Observed
Antibiotic prophylaxis	1 (10%)	Incorrect timing (>60’ from the incision)
		Failure to administer the second dose required by the procedure in relation to the length of the operation
Hair removal	0	
Pre-operative skin antisepsis	7 (70%)	Excessive quantity of antiseptic
		Antiseptic dried with a fabric (sterile)
		Not centrifugal technique
Doors closed during operation	1 (10%)	Doors closed for less than 2/3 of the time
Crowding of operating room	10 (100%)	Entrance of personnel not related to the operation (from 1 to 7 people, mean 4)
Asepsis of final medication	1 (10%)	Antiseptic dried with a fabric (sterile)
Presence of personal objects in the operating room	3 (30%)	Presence of jackets, waist pack

**Table 5:** Compliance to best practices. Observations regarding personnel.

Procedure	Non-Compliance to Best Practice		
	Personnel	N. (%)	Non-Compliance Observed
Operating room attire	Surgeons and scrub nurses of the team (38)	4 (10.5%)	Cap not completely covering the hair
	Other personnel related to the operation (46)	19 (41.3%)	Mask not completely covering the nose and mouth
	Personnel not related to the operation (38)	20 (52.6%)	Use of personal clothing (personal headwear and jackets, neck warmer) (14.8%)
	Total (122)	43 (35.2%)	
Surgical hand-washing and sterile clothing	Surgeons and scrub nurses (38)	7 (18.4%)	1 (2.6%) not performed
			6 (15.8%) duration < 3’
Hand hygiene of staff after removal of sterile gloves	Surgeons (24)	19 (79.2%)	Not performed
	Scrub nurses (14)	3 (21.4%)	
	Total (38)	24 (61.5%)	
Hand hygiene of anaesthetist before contact with patient	Anaesthetists (13)	13 (100%)	6 (46.2%) never performed
			7 (53.8%) only sometimes

## References

[1] Marchi M., Pan A., Gagliotti C., Morsillo F., Parenti M., Resi D., Moro M.L., Sorveglianza Nazionale Infezioni in Chirurgia (SNICh) Study Group (2014). The Italian national surgical site infection surveillance programme and its positive impact, 2009 to 2011. Euro Surveill..

[2] World Health Organization (2016). Global Guidelines for the Prevention of Surgical Site Infection.

[3] National Institute for Health and Care Excellence (2017). Surgical Site Infections: Prevention and Treatment. NICE Clinical Guideline CG74.

[4] Anderson D.J., Podgorny K., Berríos-Torres S.I., Bratzler D.W., Dellinger E.P., Greene L., Nyquist A.C., Saiman L., Yokoe D.S., Maragakis L.L. (2014). Strategies to prevent surgical site infections in acute care hospitals: 2014 Update. Infect. Control Hosp. Epidemiol..

[5] Awad S.S. (2012). Adherence to surgical care improvement project measures and post-operative surgical site infections. Surg. Infect..

[6] World Health Organization (2009). Guidelines for Safe Surgery 2009: Safe Surgery Saves Lives.

[7] Centers for Disease Control and Prevention (2003). Guidelines for environmental infection control in health-care facilities: Recommendations of CDC and the Healthcare Infection Control Practices Advisory Committee (HICPAC). MMWR.

[8] Allen G. (2014). Implementing AORN recommended practices for environmental cleaning. AORN J..

[9] ISPESL—Istituto Superiore per la Prevenzione e la Sicurezza sul Lavoro Linee Guida Sugli Standard Di Sicurezza E Di Igiene Del Lavoro Nel Reparto Operatorio. https://www.lisaservizi.it/sites/default/files/old_sitefile/20150820174253-ISPESL-LG-SaleOperatorie.pdf.

[10] Link T., Kleiner C., Mancuso M.P., Dziadkowiec O., Halverson-Carpenter K. (2016). Determining high touch areas in the operating room with levels of contamination. Am. J. Infect. Control.

[11] National Health Service—Health Technical Memorandum (1994). Ventilation in Healthcare Premises: Management Policy.

[12] C.CLIN-Ouest (1999). Recommandations Pour les Contrôles D’environnement Dans les Établissements de Santé.

[13] C.CLIN Sud-Ouest (2016). Surveillance Microbiologique de L’environnement Dans les Établissements de Santé.

[14] SNLG—Sistema Nazionale Linee Guida (2011). Antibioticoprofilassi Perioperatoria Nell’adulto. http://www.snlg-iss.it/cms/files/LG_AntibioticoP_Unico_2008.pdf.

[15] Centers for Disease Control and Prevention (2014). Draft Guideline for the Prevention of Surgical Site Infection.

[16] World Health Organization (2009). Guidelines on Hand Hygiene in Health Care: First Global Patient Safety Challenge Clean Care is Safer Care.

[17] Bonadonna L., Biancesco R., Brunetto B., Coccia A.M., De Gironimo V., Della Libera S., Fuselli S., Gucci P.M.B., Iacovacci P., Lacchetti I. (2013). Strategie di Monitoraggio Dell’inquinamento di Origine Biologica Dell’aria in Ambiente Indoor.

[18] Lenzi D., Spataro G., Nante N., Messina G., Vonci N. (2017). Air contamination in operating rooms: Is it better to follow the guidelines or the experience?. Eur. J. Public Health.

[19] Noguchi C., Koseki H., Horiuchi H., Yonekura A., Tomita M., Sunagawa S., Osaki M. (2017). Factors contributing to airborne particle dispersal in the operating room. BMC Surg..

[20] Silva P. (2014). The right skin preparation technique: A literature review. J. Perioper. Pract..

[21] Loison G., Troughton R., Raymond F., Lepelletier D., Lucet J., Avril C., Birgand G., ARLIN Working Group (2017). Compliance with clothing regulations and traffic flow in the operating room: A multi-centre study of staff discipline during surgical procedures. J. Hosp. Infect..

[22] Balocco C., Petrone G., Cammarata G., Vitali P., Albertini R., Pasquarella C. (2014). Indoor Air Quality in a Real Operating Theatre under Effective Use Conditions. J. Biomed. Sci. Eng..

[23] Birgant G., Saliou P., Lucet J.C. (2015). Influence of staff behaviour on infectious risk in operating rooms: What is the evidence?. Infect. Control Hosp. Epidemiol..

[24] Pryor F., Messmer P.R. (1998). The effect of traffic patterns in the OR on surgical site infections. AORN J..

[25] Andersson A., Bergh I., Karlsson J., Eriksson B.I., Nilsson K. (2012). Traffic flow in the operating room: An explorative and descriptive study on air quality during orthopedic trauma implant surgery. Am. J. Infect. Control.

[26] Pasquarella C., Sansebastiano G.E., Albertini R., Saccani E., Fanti M., Dall’Aglio P., Vitali P., Marenghi P., Signorelli C. (2006). Laminar air flow (LAF) ventilation in operating room: The need for a control system to ensure the usefulness of its installation. J. Hosp. Infect..

[27] Scaltriti S., Cencetti S., Rovesti S., Marchesi I., Bargellini A., Borella P. (2007). Risk factors for particulate and microbial contamination of air in operating theatres. J. Hosp. Infect..

[28] Wan G.H., Chung F.F., Tang C.S. (2011). Long-term surveillance of air quality in medical center operating rooms. Am. J. Infect. Control.

[29] Agenzia Sanitaria Regionale Dell’emilia-Romagna—Area di Programma Rischio Infettivo (2005). Dossier n. 116/2005—Audit Delle Misure di Controllo Delle Infezioni Postoperatorie in Emilia-Romagna.

